# Resuscitation with polymeric plasma substitutes is permissive for systemic inflammatory response syndrome and sepsis in multiply injured patients: a retrospective cohort study

**DOI:** 10.1186/s40001-016-0227-8

**Published:** 2016-10-13

**Authors:** Kai Sprengel, Hanspeter Simmen, Clément M. L. Werner, Simon Sulser, Michael Plecko, Catharina Keller, Ladislav Mica

**Affiliations:** 1 Division of Trauma Surgery, University Hospital of Zürich, Rämistrasse 100, 8091 Zurich, Switzerland; 2 Institute of Anesthesiology, University Hospital of Zürich, 8091 Zurich, Switzerland; 3 LVR Klinik Köln, 51109 Cologne, Germany

**Keywords:** Multiple trauma, Systemic inflammatory response syndrome, Sepsis, Hydroxyethyl starch derivatives

## Abstract

**Objective:**

Multiple trauma is often accompanied by systemic inflammatory response syndrome (SIRS). The aim of this study was to investigate the impact of polymeric plasma substitutes on the development of SIRS or sepsis.

**Methods:**

We included 2969 patients aged ≥16 years with an Injury Severity Score (ISS) >16 in this study. The sample was subdivided into three groups: patients who did not receive colloids and those who received <5L colloids and >5L colloids within the first 48 h. Data were analyzed using IBM SPSS^®^ for Windows version 22.0; analysis of variance was used for continuous normally distributed data and Kruskal–Wallis test for categorical data. The predictive quality of colloid treatment was analyzed using the receiver operating characteristic (ROC) curves. Independent predictively was analyzed by binary logistic regression. Data were considered significant if *P* < 0.05. Data are presented as the mean ± standard deviation.

**Results:**

The SIRS score increased with the amount of colloid used (1.9 ± 1.4 vs. 2.4 ± 1.2 vs. 3.2 ± 0.9; *P* < 0.001). However, the predictive quality was low, with an area under the ROC of 0.693 for SIRS and 0.669 for sepsis (*P* < 0.001). Binary logistic regression revealed colloids as an independent factor for the development of SIRS and sepsis (odds ratios: SIRS 3.325 and sepsis 8.984; *P* < 0.001).

**Conclusion:**

Besides other factors, colloids have a significant permissive effect and are independent predictors for the development of SIRS and sepsis in multiply injured patients.

*Trial registration ‘Retrospektive Analysen in der Chirurgischen Intensivmedizin’* No. St. V. 01-2008

## Background

The most frequent cause of death in the young and productive adult population is trauma. Bleeding, surgery, and coagulopathy are the main killers of severely injured patients [1, 2]. The more severely a patient is injured, the more they tend to bleed and develop systemic inflammatory response syndrome (SIRS) [3]. Besides emergency surgical interventions, efficient infusion therapy in severely injured patients is the key method of improving the survival of patients. According to Advanced Trauma Life Support (ATLS) guidelines, the initial volume therapy should be administered in a balanced way with further ongoing saline or transfusion therapy according to the patient’s physiological state with a permissive hypotension. The symptoms of SIRS in severe injury resemble a systemic disease. During the last decade, hydroxyethyl starch derivates (HES) and other colloids have been extensively postulated as therapeutic agents to prevent capillary leakage during SIRS and to influence blood coagulation [4–6]. Overactivation of the immune system during SIRS leads to its depression through the compensatory anti-inflammatory response syndrome (CARS). In this phase, multiply injured patients are highly susceptible to infections, which increase their mortality and hospitalization. Positive immunomodulation by an appropriate infusion therapy in multiply injured patients could be very useful for improving the survival and outcome of these patients. Hospitalization could be shortened, leading to a decrease in treatment costs. The colloids included in this study are both HES and modified gelatin. The absence of clear infusion protocols for colloidal plasma expanders may lead to involuntary mixtures of infused colloids. In this retrospective cohort study, we asked how colloids influence the development of SIRS and sepsis in patients with multiple traumas, apart from other factors with a significant permissive effect on the development of SIRS and sepsis.

## Methods

### Patient sample

In this retrospective cohort study, we included 2969 patients with severe injuries admitted to the trauma bay of the University Hospital of Zürich (Switzerland) during the period 1996–2011. The data from 120 patients were incomplete and excluded from this study. The inclusion criteria were an Injury Severity Score (ISS) >16 points, age ≥16 years, and admission within at least 24 h of incurring the severe injury. The patient sample was subdivided into three groups (Table 1) according to the use or otherwise of colloids. All patient data were collected retrospectively. The observation period was 30 days maximally or until the discharge of the patient. The data were retrieved from patient records with the approval of the local institutional review board (IRB) according to the University of Zürich IRB guidelines and the World Medical Association Declaration of Helsinki. The study was conducted according to our institutional guidelines for good clinical practice (Ethics Committee of the University Hospital of Zürich Permission: *‘Retrospektive Analysen in der Chirurgischen Intensivmedizin’* No. St. V. 01-2008). No individual consent was required. The data were not age or sex matched.

**Table 1:** The characteristics of the patient sample at admission for those not receiving colloids vs. receiving colloids <5L/48 h vs. colloids >5L/48 h

At admission	No colloids	Colloids <5L/48 h	Colloids >5L/48 h	*P* value
Age (a)	46.9 ± 20.1	43.7 ± 19.2	37.4 ± 16.3	<0.001*
Gender (male/female)	1211/448	618/240	357/95	<0.001^‡^
AIS head	3.0 ± 2.0	2.5 ± 2.0	3.2 ± 1.9	<0.001*
AIS face	0.5 ± 1.0	0.6 ± 1.1	0.7 ± 1.1	<0.001^†^
AIS thorax	1.5 ± 1.7	1.7 ± 1.7	2.0 ± 1.7	<0.001*
AIS abdomen	1.0 ± 1.7	1.0 ± 1.6	1.4 ± 1.9	<0.001^†^
AIS spine	0.7 ± 1.3	0.9 ± 1.4	0.9 ± 1.5	<0.001^†^
AIS extremities	1.2 ± 1.4	1.5 ± 1.5	1.8 ± 1.5	<0.001*
AIS pelvis	0.5 ± 1.1	0.6 ± 1.2	0.7 ± 1.3	0.010^†^
AIS soft tissue	0.4 ± 0.8	0.6 ± 0.8	0.6 ± 0.8	<0.001^†^
ISS	28.1 ± 14.5	26.8 ± 13.4	33.8 ± 13.4	<0.001*
NISS	38.5 ± 17.8	34.6 ± 15.1	44.1 ± 15.1	<0.001*
GCS	8.5 ± 5.5	9.8 ± 5.3	6.7 ± 5.1	<0.001*
Base excess (mEq/L)	−3.9 ± 6.2	−3.3 ± 4.3	−4.9 ± 4.6	<0.001*
Lactate (mmol/L)	3.3 ± 2.9	2.7 ± 2.0	3.1 ± 2.3	<0.001*
Hematocrit (%)	33.3 ± 9.0	34.6 ± 7.4	31.8 ± 8.4	<0.001*
Hemoglobin (g/dL)	11.3 ± 4.7	11.6 ± 2.5	10.7 ± 3.0	<0.001*
Prothrombin time (%)	77.7 ± 23.5	82.0 ± 19.7	75.5 ± 21.8	<0.001*
Leukocytes (10^3^/μL)	17.8 ± 5.6	13.4 ± 5.8	13.2 ± 5.9	0.025^†^
APACHE II	15.5 ± 9.8	12.6 ± 7.2	16.8 ± 7.4	<0.001*
Erythrocytes (U)	15.0 ± 15.0	0.8 ± 2.5	4.9 ± 10.6	<0.001*
Platelets (U)	0.6 ± 3.6	1.6 ± 5.4	9.7 ± 21.1	<0.001*
FFP (U)	0.7 ± 4.0	2.8 ± 7.2	12.5 ± 15.9	<0.001*

The precise injury pattern and the baseline physiological parameters at admission are shown. *GCS* Glasgow Coma Scale, *FFP* fresh frozen plasma

* ANOVA

^**†**^Kruskal–Wallis

^**‡**^ *χ* ^2^ Significant if *P* < 0.05

### Diagnostic protocol

Unstable patients underwent resuscitative procedures according to the ATLS guidelines of the American College of Surgeons. Hemodynamically stable patients received diagnoses according to clinical findings or whole-body computed tomography (CT) in uncertain situations. Hemodynamically unstable patients received focus-oriented diagnostics with immediate problem solving according to the ATLS guidelines.

### Primary care

The treatment of all patients admitted was according to the ATLS guidelines and the previously assessed trauma management protocol, after appropriate indications had been identified [7, 8].

### Scoring systems

The overall physiological impairment was evaluated from the Acute Physiology and Chronic Health Evaluation (APACHE II) score of the patient at admission [9]. The ISS and the New Injury Severity Scale (NISS) were used to define the severity of trauma [10, 11]. The Abbreviated Injury Scale (AIS; 2005 version) was used to describe injuries in specific anatomical regions.

### Laboratory parameters

Blood lactate levels, pH, and hematocrits were measured at intervals using a blood gas analyzer (ABL800 Flex, Radiometer, Thalwil, Switzerland). The prothrombin time was measured using a standardized method [12].

### Transfusion resuscitation of multiply injured patients

Infusion and transfusion therapies for multiply injured patients were applied according to damage control resuscitation criteria [13] and the guidelines of the University Hospital of Zurich [14].

### Plasma substitutes

The only plasma substitutes (colloids) used were Physiogel balanced (succinylated gelatin, 23.2 [kDa], B. Braun Medical, Sempach, Switzerland), Voluven (hydroxyethyl starch 130/0.4) 6 % (Fresenius Kabi, Bad Homburg, Germany), and Tetraspan (hydroxyethyl starch 130/0.4) 6 % (B. Braun Medical).

### Assessment of SIRS and sepsis

The worst values for leukocyte count, respiratory rate, heart rate, and temperature were taken to determine the SIRS score each day [15]. SIRS was measured during the first 30 days after admission or as long as the patients were hospitalized. Sepsis was defined as an SIRS score ≥2 with an infectious focus.

### Statistical analysis

Data are presented as the mean ± standard deviation for continuous variables and as percentages for categorical variables. Cases with an incomplete data set were discarded from this study (*n* = 52). Two-tailed Kolmogorov–Smirnov tests were used for testing normality and, if *P* < 0.05, the data were considered to be normally distributed. The data for the groups were compared using a *χ* ^2^ test and a Kruskal–Wallis test for categorical data and one-way analysis of variance (ANOVA) for continuous data. If a Kolmogorov–Smirnov test showed *P* > 0.05, Mann–Whitney non-parametric *U* test was used for continuous data. Results were considered significant if *P* < 0.05. The predictive quality for SIRS and sepsis of colloids was reported as the area under the receiver operator characteristic (ROC) curve. The entire amount of infused colloids was used as a predictor for SIRS and sepsis. Odds ratios (ORs) were calculated for categorical data. Independent predictivity was analyzed using binary logistic regression with the Hosmer–Lemeshow test for the goodness of fit; good if *P* > 0.05. Data were analyzed using IBM SPSS Statistics for Windows software (version 22.0; IBM Corp., Armonk, NY, USA).

## Results

### Patient sample

The group of patients not receiving colloids was significantly larger than the group that received colloids <5L/48 h and >5L/48 h (1659 vs. 858 vs. 452, *P* < 0.001). There were significantly more male than female patients in all three groups (*P* < 0.001) (Table 1). The patients who did not receive colloids were significantly older than those who received colloids <5L/48 h and >5L/48 h [46.9 ± 20.1 vs. 43.7 ± 19.2 vs. 37.4 ± 16.3 (a); *P* < 0.001; Table 1]. Patients receiving colloids >5L/48 h were significantly more severely injured. Interestingly, patients receiving colloids <5L/48 h had the lowest trauma load (ISS: 28.1 ± 14.5 vs. 26.8 ± 13.4 vs. 33.8 ± 13.4, *P* < 0.001; NISS: 38.5 ± 17.8 vs. 34.6 ± 15.1 vs. 44.1 ± 15.1; *P* < 0.001; Table 1). The lactate levels [3.3 ± 2.9 vs. 2.7 ± 2.0 vs. 3.1 ± 2.3 (mmol/L); *P* < 0.001; Table 1] and base excess [–3.9 ± 6.1 vs. –3.3 ± 4.3 vs. –4.9 ± 4.6 (m Eq/L); *P* < 0.001; Table 1] were significantly elevated in patients from the group who received colloids >5L/48 h compared with the levels and base excess in patients from the group not receiving colloids and those in the group receiving colloids <5L/48 h. Calculation of the APACHE II score showed similar results (15.5 ± 9.8 vs. 12.6 ± 7.2 vs. 16.8 ± 7.4; *P* < 0.001; Table 1); the value was significantly elevated in patients from the group who received colloids >5L/48 h compared with that in patients from the group not receiving colloids and those from the group receiving colloids <5L/48 h.

### Analysis of SIRS, infection, and sepsis

The SIRS score at admission was significantly elevated in patients from the groups receiving colloids (2.1 ± 1.2 vs. 2.2 ± 1.1 vs. 2.6 ± 1.1; *P* < 0.001; Table 2). An increase over time in the SIRS score was observed in these patients (1.9 ± 1.4 vs. 2.4 ± 1.2 vs. 3.2 ± 0.9; *P* < 0.001; Table 2); however, a maximum was reached more slowly in patients from the group receiving colloids [2.2 ± 3.6 vs. 3.1 ± 4.4 vs. 5.9 ± 5.7 (d); *P* < 0.001; Table 2]. The rates of sepsis increased according to increasing colloid use (10 vs. 16 vs. 36 %; *P* < 0.001; Table 2). However, the onset of sepsis was later according to the use of colloids [7.9 ± 7.1 vs. 6.4 ± 5.4 vs. 9.1 ± 5.7 (d); *P* < 0.001; Table 2].

**Table 2:** The development of SIRS and sepsis in the patient sample

	No colloids	Colloids <5L/48h	Colloids >5L/48h	*P* value
SIRS admission	2.1 ± 1.2	2.2 ± 1.1	2.6 ± 1.1	<0.001^‡^
SIRS maximum	1.9 ± 1.4	2.4 ± 1.2	3.2 ± 0.9	<0.001*
SIRS day of maximum	2.2 ± 3.6	3.1 ± 4.4	5.9 ± 5.7	<0.001*
Sepsis (% of each group)	10	16	36	<0.001^‡^
Day of sepsis onset	7.9 ± 7.1	6.4 ± 5.4	9.1 ± 5.7	<0.001^‡^
Septic shock (% of each group)	3	2	9	<0.001^‡^

Patients not receiving colloids vs. receiving colloids <5L/48h vs. colloids >5L/48h are compared. The sequence of SIRS development, sepsis, and the onset of sepsis and septic shock are shown in the investigated patient sample

* ANOVA

^**‡**^Kruskal–Wallis

Significant if *P* < 0.05

Binary logistic regression revealed the application of colloids to be an independent factor in the development of SIRS (Wald: 174.229; OR 3.325; *P* < 0.001) and sepsis (Wald: 108.989; OR 8.984; *P* < 0.001). However, EC, TC, and FFP revealed SIRS and sepsis to be an independent predictors for multiply injured patients (Table 3A). Interestingly, the onset was earliest in patients from the group receiving colloids <5L/48 h. ROC analysis showed the highest predictive power for SIRS (AUC 0.69; *P* < 0.001, CI 95 %, 0.653, 0.733; OR 3.33), followed by sepsis (AUC 0.669; *P* < 0.001; CI 95 %, 0.637, 0.706; OR 2.72) (Table 3B.).

**Table 3:** (A) The binary logistic regression analysis of the patient sample revealed that the infusion of colloids within the first 48 h after trauma is an independent predictor for the development of SIRS and sepsis. Hosmer–Lemeshow test, *P* < 0.001 for SIRS and *P* < 0.001 for sepsis. (B) ROC curve of the patient sample

Binary logistic regression	Wald	Odds	*P* value
(A) The binary logistic regression analysis of the patient sample			
SIRS (colloids)	174.229	3.325	<0.001
Sepsis (colloids)	108.989	8.984	<0.001
SIRS (EC)	39.242	1.955	<0.001
Sepsis (EC)	69.910	1.848	<0.001
SIRS (platelets)	6.303	0.972	0.012
Sepsis (platelets)	0.005	0.998	0.944
SIRS (FFP)	4.335	0.942	0.037
Sepsis (FFP)	10.447	1.217	0.001

ROC	AUC	*P* value
(B) Predictive quality depicted by AUC of the corresponding ROC		
SIRS (colloids)	0.693	<0.001
Sepsis (colloids)	0.669	<0.001
SIRS (EC)	0.539	<0.001
Sepsis (EC)	0.821	<0.001
SIRS (platelets)	0.501	<0.001
Sepsis (platelets)	0.677	<0.001
SIRS (FFP)	0.512	<0.001
Sepsis (FFP)	0.807	<0.001

*AUC* area under the curve, *FFP* fresh frozen plasma

Significant if *P* < 0.05

### Outcome of the patient sample

Interestingly, the overall mortality was significantly lower in the colloid-treated groups (40 vs. 12 vs. 20 %; *P* < 0.001; Table 4A) than in the group not treated with colloids. The hospitalization time [13.4 ± 19.5 vs. 19.7 ± 14.7 vs. 28.0 ± 22.5 (d); *P* < 0.001; Table 5A], intensive care unit (ICU) stay [5.9 ± 9.0 vs. 8.9 ± 9.4 vs. 18.3 ± 13.1 (d); *P* < 0.001; Table 5A], and ventilator-assisted ICU treatment [3.5 ± 6.8 vs. 5.3 ± 7.4 vs. 13.1 ± 10.4 (d); *P* < 0.001; Table 4A] were significantly increased in patients from the groups treated with colloids. However, patients treated with colloids that eventually died survived longer than those who were not treated with colloids [1.9 ± 4.4 vs. 6.7 ± 9.2 vs. 12.7 ± 15.0 (d); *P* < 0.001; Table 4A]. In the binary logistic regression, polymeric plasma substitutes only influenced the time spent in the hospital (Wald: 7.205; OR 0.767; *P* = 0.007; Table 4B), the number of respirator-associated days in the ICU (Wald: 5.065; OR 1.154; *P* = 0.024; Table 4B), and the time of death (Wald: 8.039; OR 1.142; *P* = 0.005; Table 4B). The application of colloids in multiply injured patients is not an independent predictor of death, as shown by the binary logistic regression (Wald: 0.000; OR 1.000; *P* = 1.000; Table 4B).

**Table 4:** The outcome (A) of the patient sample with its binary logistic regression (B) to detect colloids as an independent factor for an adverse outcome under severe injury conditions

Outcome	No colloids	Colloids <5L/48h	Colloids >5L/48h	*P* value
(A) The outcome of the patient sample				
Hospitalization (d)	13.4 ± 19.5	19.7 ± 14.7	28.0 ± 22.5	<0.001*
ICU (d)	5.9 ± 9.0	8.9 ± 9.4	18.3 ± 13.1	<0.001*
Respirator (d)	3.5 ± 6.8	5.3 ± 7.4	13.1 ± 10.4	<0.001*
Death [d]	1.9 ± 4.4	6.7 ± 9.2	12.7 ± 15.0	<0.001*
Death (% of each group)	40	12	20	<0.001^†^

Outcome binary logistic regression	Wald	Odds	*P* value
(B) Independent outcome parameters of colloid application			
Hospitalization (d)	7.205	0.767	0.007
ICU (d)	3.560	1.233	0.059
Respirator (d)	5.065	1.154	0.024
Death (d)	8.039	1.142	0.005
Death (% of each group)	0.000	1.000	1.000

* ANOVA

^**†**^Kruskal–Wallis

Significant if *P* < 0.05

**Table 5:** Possible co-founding factors of SIRS and sepsis

	SIRS			Sepsis		
	Wald	Odds	*P* value	Wald	Odds	*P* value
Age (a)	0.423	0.998	0.515	7.968	0.991	0.005
Gender (male/female)	0.000	0.998	0.987	0.252	1.065	0.616
AIS head	0.006	0.996	0.936	1.130	1.045	0.288
AIS face	0.096	1.020	0.757	0.695	1.043	0.405
AIS thorax	0.028	0.992	0.867	1.292	1.044	0.256
AIS abdomen	0.044	0.990	0.833	0.525	1.028	0.469
AIS spine	*7.822*	*1.149*	*0.005*	2.587	1.064	0.108
AIS extremities	*9.302*	*1.171*	*0.002*	*23.463*	*1.220*	*<0.001*
AIS pelvis	*8.782*	*1.211*	*0.003*	2.823	0.924	0.093
AIS soft tissue	0.008	0.993	0.930	2.961	1.118	0.085
ISS	0.067	1.003	0.795	0.071	1.002	0.790
NISS	3.112	1.012	0.078	0.601	0.996	0.438
GCS	*20.401*	*0.907*	*<0.001*	*7.397*	*0.954*	*0.007*
Base excess (m Eq/L)	*5.449*	*0.956*	*0.020*	*30.932*	*0.907*	*<0.001*
Lactate (mmol/L)	*4.136*	*0.928*	*0.042*	*15.893*	*0.879*	*<0.001*
Hematocrite (%)	1.230	0.971	0.267	0.007	1.002	0.934
Hemoglobin (g/dL)	0.455	1.055	0.500	0.029	0.988	0.864
Prothrombin time (%)	0.777	1.003	0.378	*4.785*	*1.006*	*0.029*
Leukocytes (10^3^/μL)	*7.267*	*1.034*	*0.007*	3.344	1.018	0.067
APACHE II	1.091	0.984	0.296	*10.359*	*0.959*	*0.001*

Significant results are highlighted in italics. Hosmer–Lemeshow* P* = 0.001 fors SIRS and* P* = 0.2 for sepsis

### Analysis of co-founding factors of SIRS and sepsis

The analysis revealed that in both groups, SIRS and sepsis, the GCS, base excess, and lactate played a significant permissive role for the development of SISR and sepsis in multiply injured patients (Table 5). The AIS spine, extremities, and pelvis as well as the prothrombin time, leukocyte counts, and APACHE II score had a significant effect on the development of SIRS and sepsis (Table 5.).

## Discussion

There is no doubt that polymeric saccharides such as HES can effectively expand blood volume and improve survival in patients with multiple injuries. The analysis submitted to the US Food and Drug Authority in the early 1970s would not be considered adequate to detect the possible side effects of HES in the present day. Since then, an increasing number of publications with concerns about HES, but with only partially selective outcome reporting (only positive outcomes) have been published [16–19]. In this retrospective cohort study, the focus was set on the application of polymeric plasma substitutes in the context of severe injury. Therefore, the question was asked how these substances influence the immunity system in multiple trauma patients. Patients who received more polymeric plasma substitute suffered significantly more severe SIRS and sepsis. Certainly, there are a lot of confounders for the development of SIRS and sepsis in multiply injured patients, but one key factor is the use of polymeric plasma substitutes, as supported by the highly significant binary logistic regression analysis (Table 3). Therefore, in this study, it may be concluded that besides other factors, polymeric plasma substitutes contribute significantly to the development of SIRS in multiply injured patients. However, the impact of injury severity on the development of SIRS and sepsis also plays a pivotal role [3]. Overactivation of the immune cells for the clearance of destroyed tissue might be the reason. The products used in this study vary over almost two decades; however, the one thing they all have in common is that they are all polymers. Polymeric substances might have pharmacologic effects on the reticulo-endothelial system (RES). There is growing evidence that polymeric plasma substitutes may inhibit the RES by inhibition of cytokine secretion [20]. The question of whether polymeric plasma expanders influence the RES was asked in the late 1980s. The application of HES reduced the phagocytic activity of RES; however, the data were unclear and lacked robust statistical analysis [21, 22]. Therefore, a partial immunosuppressive effect for polymeric plasma substitutes must be postulated. This seems to be reflected in the ORs obtained by the binary logistic regression, 3.325 for SIRS and 8.984 for sepsis (Table 3). Certainly, a higher trauma load in the patient sample receiving more polymeric plasma substitutes is obtained; therefore the trauma load and other factors also contribute to the development of SIRS and sepsis [3], which is indirectly reflected by the small AUC. How brain injury is involved in both SIRS and sepsis development remains speculative. The impaired blood–brain barrier under trauma conditions may release microglial cytokines to influence the immune system systemically. At this time, little is known about the influence of sugar polymers on immune physiology in humans; however, these data show that indications for their use in patients with multiple traumas should be clarified. The mixed outcomes might also be partly caused by the higher ISS at admission, but the polymeric plasma substitutes also seem to have a significant influence on pulmonary function in multiple trauma patients, as reflected by the ventilator-associated days in the ICU, the hospitalization duration, and the time of death, somehow in a paradox manner (Table 4). Interestingly, in this patient sample, the mortality was significantly lower in the group receiving colloids >5L/48 h. The ISS, APACHE II, and all other parameters except for age were significantly higher at admission for this patient group. Age was <40 years (37.4 ± 16.3 [a]). Whether such a significant break point for multiply injured patients occurs at about the age of 40 years remains speculative; however, age appears to play a pivotal role in the pathophysiology of multiply injured patients.

## Limitations

A limitation, and a possible source of bias, is the changing fluid resuscitation protocols over the study period, which could make the interpretation less reliable. The chosen selection criteria might counteract this bias.

## Conclusions

Polymeric plasma substitutes should be applied to multiply injured patients in a more tailored fashion, because besides many other factors these plasma substitutes might permissively affect SIRS and sepsis. However, patient survival might be positively influenced by the application of polymeric plasma substitutes, as depicted in this sample.

## Abbreviations

AIS: Abbreviated Injury Scale
ANOVA: analysis of variance
APACHE II: acute physiology and chronic health evaluation
ATLS: advanced trauma life support
AUC: area under the curve
CARS: compensatory anti-inflammatory response syndrome
HES: hydroxyl ethyl starch
IBM^®^: International Business Machines Corporation^®^
IRB: Institutional Review Board
ISS: Injury Severity Score
NISS: New Injury Severity Score
OR: odds ratio
RES: reticulo-endothelial system
ROC: receiver operating curve
SIRS: systemic inflammatory response syndrome
SPSS: Statistical Package for the Social Sciences^®^
